# *Semi-Rolled Leaf2* modulates rice leaf rolling by regulating abaxial side cell differentiation

**DOI:** 10.1093/jxb/erw029

**Published:** 2016-02-11

**Authors:** Xiaofei Liu, Ming Li, Kai Liu, Ding Tang, Mingfa Sun, Yafei Li, Yi Shen, Guijie Du, Zhukuan Cheng

**Affiliations:** ^1^State Key Laboratory of Plant Genomics and Center for Plant Gene Research, Institute of Genetics and Developmental Biology, Chinese Academy of Sciences, Beijing 100101, China; ^2^Institute of Agricultural Sciences in Jiangsu Coastal Areas, Yancheng 224002, China

**Keywords:** Abaxial side, cell differentiation, leaf development, leaf rolling, rice, SRL2.

## Abstract

Moderate leaf rolling maintains the erectness of leaves and minimizes the shadowing between leaves which is helpful to establish ideal plant architecture. Here, we describe a *srl2* (*semi-rolled leaf2*) rice mutant, which has incurved leaves due to the presence of defective sclerenchymatous cells on the abaxial side of the leaf and displays narrow leaves and reduced plant height. Map-based cloning revealed that *SRL2* encodes a novel plant-specific protein of unknown biochemical function. *SRL2* was mainly expressed in the vascular bundles of leaf blades, leaf sheaths, and roots, especially in their sclerenchymatous cells. The transcriptional activities of several leaf development-related *YABBY* genes were significantly altered in the *srl2* mutant. Double mutant analysis suggested that *SRL2* and *SHALLOT-LIKE1* (*SLL1*)/*ROLLED LEAF9* (*RL9*) function in distinct pathways that regulate abaxial-side leaf development. Hence, SRL2 plays an important role in regulating leaf development, particularly during sclerenchymatous cell differentiation.

## Highlight

*Semi-Rolled Leaf*2 encodes a novel plant-specific protein that modulates leaf rolling by regulating cell development in the abaxial sides of rice leaves.

## Introduction

Leaf shape has long been considered to be an important agronomic trait in rice. Leaf functions such as photosynthesis, respiration, and transpiration are dependent on leaf shape and its three-dimensional architecture (Govaerts *et al.*, 1996). In rice (*Oryza sativa*) and other crops cultivated at high density, moderate leaf rolling can help maintain the erectness of leaves which ultimately benefits crops growth by improving light acceptance, delaying leaf senescence, and accelerating the accumulation of dry matter. The last three leaves of super-high-yielding hybrid rice were proposed to be long, erect, narrow, rolled (V-shaped), and thick (Yuan, 1997). Therefore, identifying mutants with moderately rolled leaves and isolating genes that control leaf rolling will be beneficial for breeding crops with the desired architecture and stress tolerance.

As rice is a polymorphic crop, rice varieties and mutants exhibit two different types of transverse leaf rolling, i.e. inward (adaxial) and outward (abaxial) rolling, which are regulated by complicated developmental processes including the control of polarity establishment and cell differentiation (Bowman *et al.*, 2002; Micol and Hake, 2003). Leaf polarity establishment is controlled by both transcription factors and small RNAs (Moon and Hake, 2011). HD-ZIPIII family genes, such as *PHABULOSA* (*PHB*), *PHAVOLUTA* (*PHV*) (McConnell *et al.*, 2001), *REVOLUTE* (*REV*) (Otsuga *et al.*, 2001), and *ROLLED1* (*RLD1*) (Nelson *et al.*, 2002), determine the development of adaxial cells in leaves. By contrast, members of the KANADI family, such as *MILK-WEED POD1* (*MWP1*) (Candela *et al.*, 2008), and genes belonging to the *YABBY* (*YAB*) family, such as *YAB2* and *YAB3* (Eshed *et al.*, 2004), determine the development of abaxial cells. Two small RNAs, *trans*-acting short interfering RNAs (tasiRNA) and miR165/166, exhibit opposing polar distribution in leaf primordial tissue and are thought to establish the adaxial–abaxial axis during leaf development (Nogueira *et al.*, 2007). Recent reports have demonstrated that cytological architecture can also affect leaf blade morphology. *SEMI-ROLLED LEAF1* (*SRL1*), encoding a putative glycosylphosphatidylinositol-anchored protein, is involved in leaf rolling through inhibiting the formation of bulliform cells (Xiang *et al.*, 2012). In addition, *SHALLOT-LIKE1* (*SLL1*), which encodes a MYB transcription factor of the KANADI family, is involved in leaf rolling through regulating sclerenchyma cell differentiation (Yan *et al.*, 2008; Zhang *et al.*, 2009). Environmental factors such as water deficiency, high temperature, and sunshine may also lead to the inward rolling of leaves (Kadioglu and Terzi, 2007).

Due to its importance, many studies have been performed to characterize the genes controlling leaf rolling in rice. To date, 17 rice mutants with rolled leaves have been characterized, from which six recessive genes (*rl1*–*rl6*) were mapped to chromosomes 1, 3, 4, 7, and 12 through classical linkage analysis (Khush and Kinoshita, 1991). However, the other genes were mapped to chromosome 2 (*rl*_*(t*)_; Shao *et al.*, 2005b), chromosome 5 (*rl7* and *rl8*; Li, 1998; Shao *et al.*, 2005a), chromosome 7 (*rl11, srl1,* and *sll2*; Shi *et al.*, 2009; Xiang *et al.*, 2012; Zhang *et al.*, 2015), chromosome 9 (*rl9* and *rl10*; Luo *et al.*, 2007; Yan *et al.*, 2008; Zhang *et al.*, 2009), and chromosome10 (*rl12* and *rl14*; Fang *et al.*, 2012；Luo *et al.*, 2009) by molecular marker analysis. In addition, overexpression of *OsAGO7* results in the upward curling of rice leaves (Shi *et al.*, 2007).

Although many mutants with leaf rolling phenotypes have been characterized and their relevant genes have been identified, the molecular basis of leaf rolling in rice remains fragmented. Here, we report a *Semi-Rolled Leaf2* (*SRL2*) gene which encodes a novel plant-specific protein that functions in regulating rice leaf rolling. *SRL2* deficiency leads to abnormal formation of sclerenchymatous cells on the abaxial side of the leaf, resulting in inwardly rolled leaves. Hence, this gene might be involved in the transdifferentiation process from mesophyll cells to sclerenchymatous cells, revealing a possible mechanism underlying leaf rolling in monocot plants.

## Materials and methods

### Plant materials and growth conditions

The rice (*Oryza sativa*) semi-rolled leaf mutant *srl2* was isolated from the γ-ray-irradiated *indica* cultivar Zhongxian 3037. The F_2_ mapping population was generated from a cross between the *srl2* mutant and Nipponbare, a typical *japonica* cultivar. In the F_2_ population, plants exhibiting rolled leaves were selected for gene mapping. All plants were grown in the paddy fields of the Institute of Genetics and Developmental Biology in Beijing (China) or Sanya (Hainan Province, China) during the natural growing season.

### Map-based cloning

*SRL2* was mapped and cloned with 2 994 F_2_ mutant plants using STS molecular markers (see Supplementary Table S1 at *JXB* online). For the identification of candidate genes the corresponding DNA fragments were amplified from mutants and wild-type plants using LA-Taq (TaKaRa, http://www.takara-bio.com/) and sequenced using an Applied Biosystems 3730 sequencer (http://www.appliedbiosystems.com/).

For functional complementation, a 16.9kb genomic DNA fragment, corresponding to the entire *LOC_Os03g19520* gene, was digested from BAC clone OSJNBb0101E03 (Arizona Genomics Institute, http://www.genome.arizona.edu) using *Eco*RI and inserted into the pCAMBIA1300 vector (Cambia, http://www.cambia.org/) to generate construct pCSRL2. A control construct, pCSRL2-CK, was generated by digesting pCSRL2 with *Xho*I to remove the whole ORF of *SRL2*. The two binary plasmids were introduced into *Agrobacterium tumefaciens* EHA105 by electroporation and transformed into the *srl2* mutant plants. For RNAi analysis, a 384bp fragment comprising 316bp of the 5′ UTR and 68bp of the ORF was amplified using primers CTCGAGGTGGAGACGGGTGGTGTGTG and AGATCTGAGCTTGGGCGCAGAGCTGG. After sequencing confirmation, the PCR products were inserted into the vector pCAMBIA23A in both the forward and reverse directions to generate the construct pSRL2RNAi. This binary plasmid was then introduced into the wild-type plants (Zhongxian 3037) by *Agrobacterium* infection. Rice transformation was performed as described by Hiei *et al*. (1994).

### Microscopy

Culm segments were fixed in formalin-acetic acid-alcohol (FAA) solution (10% formaldehyde, 5% acetic acid, and 47.5% ethanol) at 4 °C overnight. After dehydration in a graded ethanol series, the samples were infiltrated and embedded in Technovit 7100 resin (Heraeus kulzer). Sections, 3 μm thick, were cut, stained with toluidine blue, and viewed under a light microscope (Leica, http://www.leica-microsystems.com). The root cell length was determined by staining the whole roots of 7-d-old seedlings with 20 μg ml^−1^ of propidium iodide in PBS, followed by examination under a fluorescent microscope (Leica). For scanning electron microscopy (SEM), the 4th leaves of the wild type and *srl2* were fixed overnight at 4 °C with 2.5% glutaraldehyde in 0.1M phosphate buffer (pH 7.4). After dehydration in a graded ethanol series and substitution with isoamyl acetate, the samples were critical-point dried, sputter coated with gold, and observed with a S-3000N scanning electron microscope (HITACHI).

### Promoter–reporter gene fusion studies

For promoter–GUS fusion studies, a 2.1-kb genomic DNA fragment that contained the promoter region of the *SRL2* gene was amplified by PCR and then subcloned into vector pCAMBIA1301 (Cambia), which resulted in a fusion of the *SRL2* promoter and the GUS reporter gene. The construct was transformed into wild-type plants as described earlier. Developing leaves of the transgenic plants were harvested and incubated in GUS reaction buffer (1mM 5-bromo-4-chloro-3-indolyl-d-glucuronide, 50mM sodium phosphate (pH 7.0), and 7% (v/v) methanol) at 37 °C overnight. The stained samples were then dehydrated in 95% ethanol until the chlorophyll was removed. For observation of the sclerenchyma in the leaf sheath, samples were hand cut with a sharp razor and mounted for microscopy. For microtome sections (8 μm), samples were prepared according to methods as described by Suzaki *et al.* (2004).

### Phylogenetic analysis

The amino acid sequences of the SRL2 homological proteins were retrieved through a database search using the total amino acid sequences of SRL2. The multiple sequence alignments were performed by Clustal_X 1.83 (Thompson *et al.*, 1997) using the parameters of Weight matrix: Gonnet; Gap opening penalty: 10.0 and Gap extension penalty: 0.10. Phylogenetic trees were constructed with the aligned protein sequences using MEGA 3.1 software (Kumar *et al.*, 2004) based on the Maximum likelihood method with parameters of the Poisson correction model, complete deletion, and bootstrap (1 000 replicates; random seed).

### Subcellular localization studies of SRL2

The coding sequence of *SRL2* was amplified by PCR and then subcloned into vector pJIT163-GFP to obtain the SRL2–GFP construct. The resultant construct was introduced into rice protoplasts and onion epidermal cells. Transfection of rice protoplasts with SRL–GFP was performed as described by Li *et al.* (2009). For the transformation of onion cells, those plasmids were bombarded into onion epidermal cells using a PDS-1000 ⁄ He particle gun (Bio-Rad). The green fluorescence was observed by confocal laser-scanning microscopy (Zeiss LSM 510 META) with an argon laser excitation wavelength of 488nm.

Onion epidermis was ground in liquid nitrogen. Total protein was extracted with protein extraction buffer (50mM TRIS–HCl at pH 7.5, 150mM NaCl, 5mM EDTA, 0.2% NP-40, 0.1% Triton X-100, and Complete protease inhibitor cocktail, Roche), usually 600 μl for 0.2g onion epidermis. The extracts were centrifuged at 16 000rpm for 15min at 4 ℃, and the supernatants were collected for Western blot analysis. Western blot analysis was performed with a monoclonal mouse anti-GFP (Abmart) primary antibody at a final dilution of 1:2 000, and with a goat pAb to mouse IgG (Abcam) second antibody at a final dilution of 1:20 000.

### *In situ* hybridization

Leaves were collected from fourth leaf stage of the wild type and fixed in FAA overnight at 4 °C. Samples were then dehydrated through a butanol series and embedded in Paraplast Plus (Sigma–Aldrich, http://www.sigmaaldrich.com/). Sections, 8 μm thick, were obtained using a Leica RM2135 microtome (Leica Biosystems, http://www.leica.com). To prepare the *SRL2* probe, a 492-bp fragment of *SRL2* cDNA was amplified using primers SRL2 T7 and SRL2 sp6 (SupplementaryTable S2). The probe was synthesized using a DIG RNA labelling kit (SP6/T7; Roche Diagnostics Ltd, http://www.roche.com) in accordance with the manufacturer’s recommendations. Pretreatment of sections, hybridization, and immunological detection were performed as described previously (Xiao *et al.*, 2009).

### Real-Time qRT-PCR

Real-time qRT-PCR analysis was performed to examine the expression pattern of *SRL2* in various tissues and at different stages of leaf growth in order to identify the transgenic lines in which *SRL2* expression was enhanced and complemented. Transcripts from genes related to leaf development that were altered in *SRL2* plants compared with the wild type were also identified. Total RNA was extracted using TRIzol solution and reverse transcribed using the Superscript Preamplification System in accordance with the manufacturer’s instructions and then analysed quantitatively on a Bio-Rad CYF96 using the real-time PCR Master Mix (TaKaRa Biotechnology Co. Ltd.). The rice *UB* gene was amplified and used as an internal standard to normalize the expression of *SRL2* and the other genes tested. The primers used to test the expression of *SRL2* and the other genes are listed in SupplementaryTable S3.

## Results

### *srl2* plants have semi-rolled leaves and reduced plant height

**Fig. 1.**
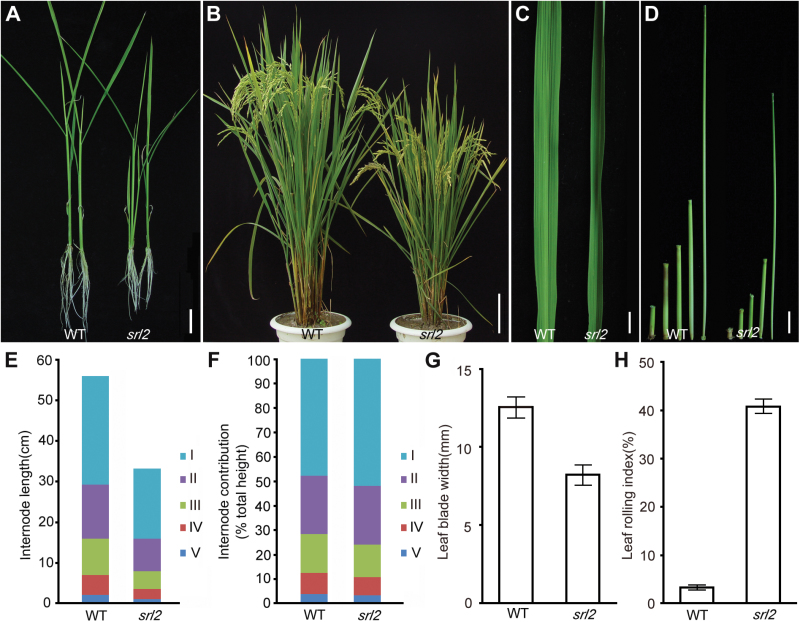
Phenotypic characterization. (A) Two-week-old wild-type and *srl2* seedlings. (B) Mature wild-type and *srl2* plants. (C) Wild-type and *srl2* leaves. (D) Wild-type and *srl2* internodes. (E) Quantification of wild-type and *srl2* internode length (mean of 15 culms ±SD). (F) Percentage of contribution of each internode to the total plant height of wild-type and *srl2* plants (mean of 15 culms ±SD). I–V, first to fifth internode. (G) Leaf width of the wild type and *srl2* (mean of 29 leaves ±SD). (H) LRI of the top two leaves of *srl2* at the heading stage. Scale bar 2cm (A, D), 10cm (B), and 1cm (C).

The *srl2* mutant was identified in a rice mutant population generated from γ-ray-irradiated *indica* rice cultivar, Zhongxian 3037. The mutant was designated *srl2* based on its semi-rolled leaves. Phenotypic observation revealed that *srl2* mutant plants had semi-uprolled leaves (Fig. 1B, C) which appeared during the seedling stage (Fig. 1A) and became more evident during plant growth. Statistical analysis revealed that, compared with wild-type leaves, the leaf rolling index (LRI) of the top second leaves of the *srl2* mutant increased by 10-fold (from 4% to 40%) at the heading stage (Fig. 1H;Supplementary Table S4). In addition to rolled leaves, *srl2* also displayed narrow leaves (wild type, 12.5±0.67mm, *n*=29; *srl2*, 8.2±0.65mm, *n*=29) (Fig. 1G;Supplementary Table S4) and reduced plant height (Fig. 1B). The dwarfism phenotype of *srl2* was exhibited throughout plant growth and development. Two-week-old mutant seedlings had reduced lengths in both the aerial parts and the roots (Fig. 1A). At the flower stage, the height of mutant plants (55.8±1.14cm, *n*=10) was 70% that of wild-type plants (79.58±1.44cm, *n*=10) (Fig. 1B;Supplementary Table S5). The reduced height resulted from uniformly shortened internodes in the mutant culms which was confirmed by comparing the length of each internode between the mutant and the wild type (Fig. 1D–F;Supplementary Table S6). In addition, *srl2* had thin culms, as well as additional morphological abnormalities including significantly reduced panicle size (SupplementaryFig. S1). Therefore, the mutation in *srl2* had multiple effects on plant growth.

### *SRL2* encodes a plant-specific protein with unknown biochemical function

**Fig. 2.**
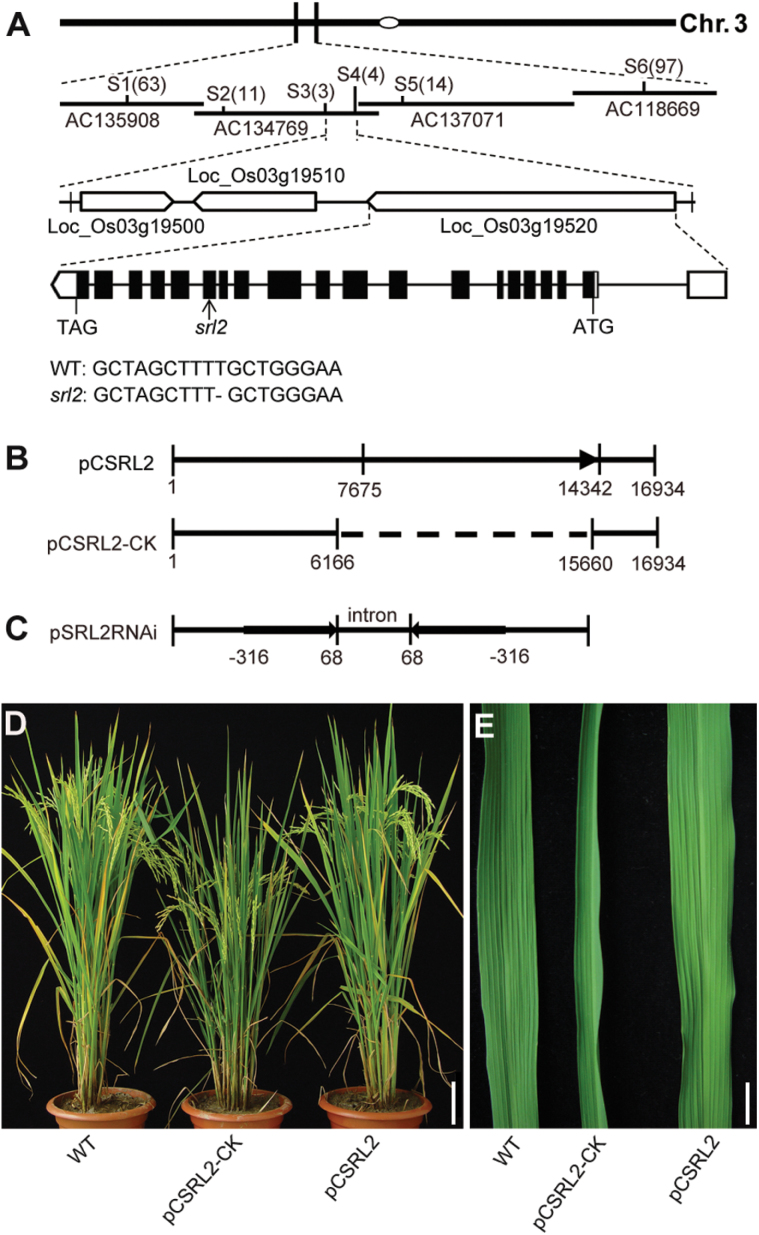
Map-based cloning of *SRL2*. (A) The *srl2* locus was mapped to a 56-kb DNA region on chromosome 3. The *SRL2* gene comprises 20 exons (boxes) and 19 introns (line). (B) Constructs used for complementary analysis; pCSRL2 and pCSRL2-CK were used to transform *srl2* plants. The dotted line in pCSRL2-CK indicates the deleted genomic region. (C) Constructs used for RNAi analysis; pSRL2RNAi was used to transform wild-type plants. (D, E) Results of complementary analysis. Scale bars=10cm (D) and 1cm (E).

Genetic analysis indicated that the rolled-leaf phenotype is controlled by a single recessive gene, with one-quarter of the F_2_ progeny displaying leaf rolling (110: 34, χ^2^ (1)=0.15, *P* >0.10). To unravel the molecular basis of this phenotype, we performed map-based cloning to isolate this gene. From the F_2_ population generated by crossing the *srl2* mutant with a *japonica* variety Nipponbare, 2 994 homozygous mutant plants were obtained and used for positional cloning. The *SRL2* locus was preliminarily mapped to the long arm of chromosome 3 between sequence-tagged site (STS) markers S1 and S6. Further mapping using adjacent STS markers indicated that the *SRL2* locus is present in BAC clone AC134769 (Supplementary Table S1); the candidate gene region was ultimately narrowed down to a 56-kb region. According to the annotations of the rice genome database (Rice Genome Annotation Project, http://rice.plantbiology.msu. edu/cgi-bin/gbrowse/rice/), this region comprises three putative genes with annotated functions. DNA sequencing revealed that one of these genes contains a mutation within the open reading frame (ORF) that co-segregated with the *srl2* phenotype. The mutation in *SRL2* in the *srl2* mutant is a deletion of a T residue in the 15th exon of *LOC_Os03g19520* (Fig. 2A), which causes a reading frame shift, resulting in the production of a predicted truncated protein. Compared with the wild type, in the deduced mutant protein, 14 amino acid residues (aa) are altered, with the exception of a 270 aa deletion (Supplementary Fig. S2).

To confirm that *LOC_Os03g19520* corresponds to the *SRL2* locus, we performed a functional complementation assay. Specifically, 16.9kb or 7.44kb of genomic DNA (with or without the entire ORF) was inserted into vector pCAMBIA 1300 to generate plasmids pCSRL2 and pCSRL2-CK, respectively (Fig. 2B). These plasmids were then transformed into the *srl2* mutant, and 46 and 34 independent transgenic lines were obtained, respectively. The mutant phenotypes were fully rescued in lines transformed with pCSRL2, but not in lines transformed with pCSRL2-CK (Fig. 2D, E). Moreover, *SRL2* knockdown transgenic plants generated by transforming an RNAi construct (Fig. 2C) into wild-type plants mimicked the *srl2* phenotypes (Supplementary Fig. S3). Thus, we conclude that *SRL2* corresponds to *LOC_Os03g19520*.

**Fig. 3.**
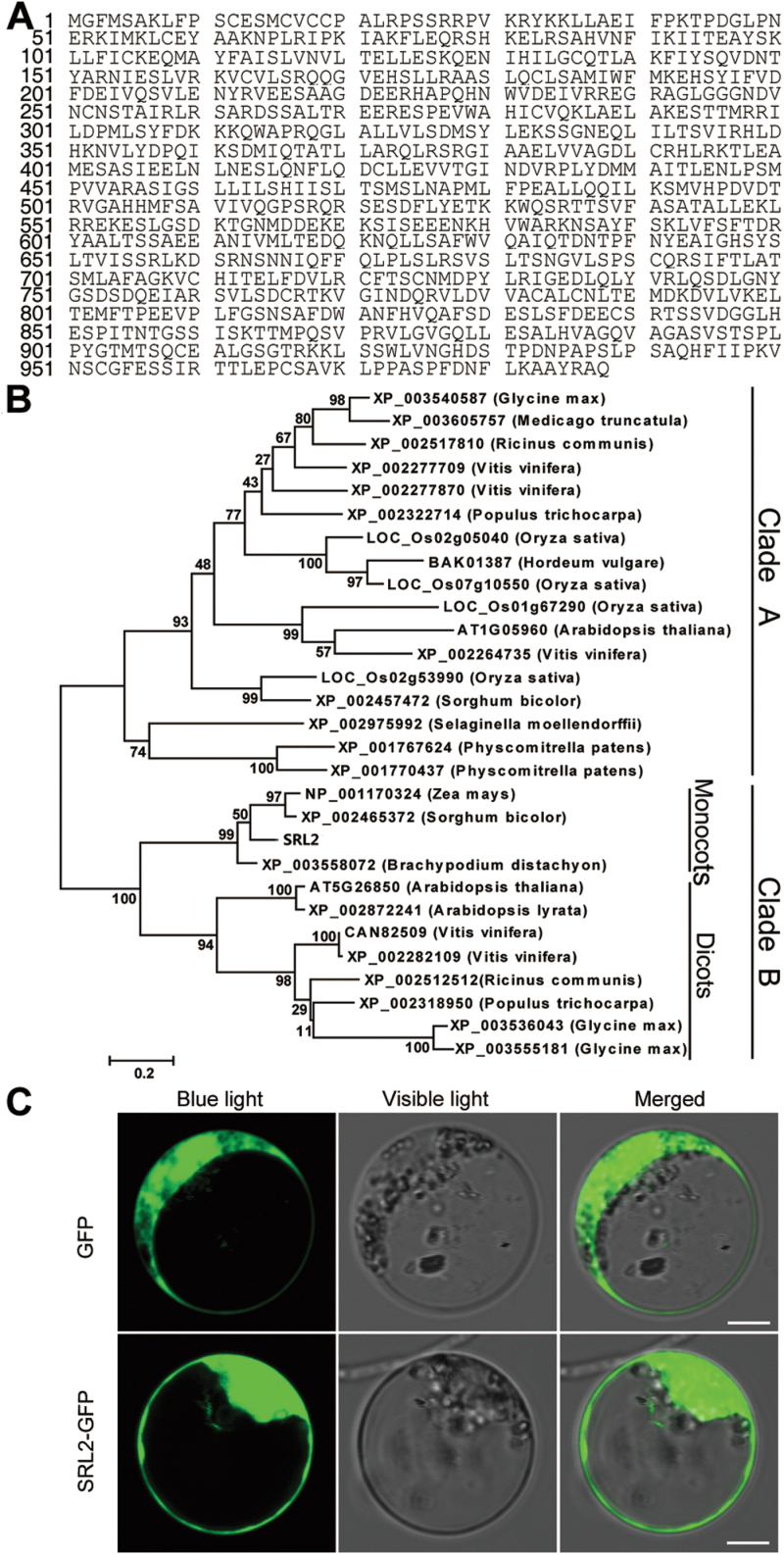
Phylogenetic analysis and subcellular localization of SRL2 protein. (A) Amino acid sequence of SRL2. (B) Phylogenetic relationships among the SRL2-like proteins. The numbers at each node represent the bootstrap support (percentage). The scale bar indicates the genetic distance based on branch length. (C) Subcellular location of the SRL2–GFP fusion protein in rice protoplasts. Scale bars=50 μm.

A KOME cDNA database search (http://cdna01.dna.affrc.go.jp/cDNA/) revealed the presence of a full-length cDNA (AK072741) corresponding to *SRL2* (*LOC_Os03g19520*). We performed RT-PCR and confirmed the sequence validation of the cDNA (AK072741). Sequence comparison between genomic DNA and cDNA revealed that *SRL2* consists of 20 exons and 19 introns (Fig. 2A) and encodes a protein with 988 amino acids (Fig. 3A). A survey of the published rice genome database (http://www.gramene.org/) revealed the existence of four additional genes designated SRL2-like genes (*LOC_Os02g05040*, *LOC_Os01g67290*, *LOC_Os02g53990*, and *LOC_Os07g10550*), which were predicted to encode proteins that share various levels of amino acid sequence identity with *SRL2*. We performed a BLASTP search against the NCBI non-redundant protein database using the SRL2 protein sequence as the query (http://blast.ncbi.nlm.nih.gov), revealing 24 SRL-like proteins from other plant species with significant homology (≥50% identity at the amino acid level) to SRL2, including monocots and dicots. Intriguingly, the existence of *SRL2*-like genes in different plant species suggests that SRL2 family members share conserved biochemical functions. However, the SRL2 sequence did not match any protein of known biochemical function in the public databases. Therefore, we propose that *SRL2* encodes a novel plant-specific protein.

To elucidate the evolutionary relationships among these SRL2-like proteins, we constructed a phylogenetic tree based on the full-length protein sequences of SRL2 and the 28 SRL2-like proteins using the Maximum likelihood method in MEGA3.1. The 29 proteins were classified into two major clades (Clade A and Clade B; Fig. 3B). While the four *SRL2* paralogous genes belong to Clade A, *SRL2* belongs to Clade B, indicating the functional diversification between *SRL2* and its paralogues in rice.

To determine the subcellular localization of SRL2, we fused the green fluorescent protein (GFP) gene to the ORF of *SRL2* driven by the 35S promoter and transformed this construct into rice protoplasts. SRL2–GFP chimeric protein, like the control, displayed a ubiquitous distribution pattern throughout the cell (Fig. 3C). We also certified the result in onion epidermis cell (Supplementary Fig. S4) and the Western blot analysis showed that the SRL–GFP was expressed normally (Supplementary Fig. S5).

### *srl2* plants have defective sclerenchymatous cells in the abaxial sides of leaves

**Fig. 4.**
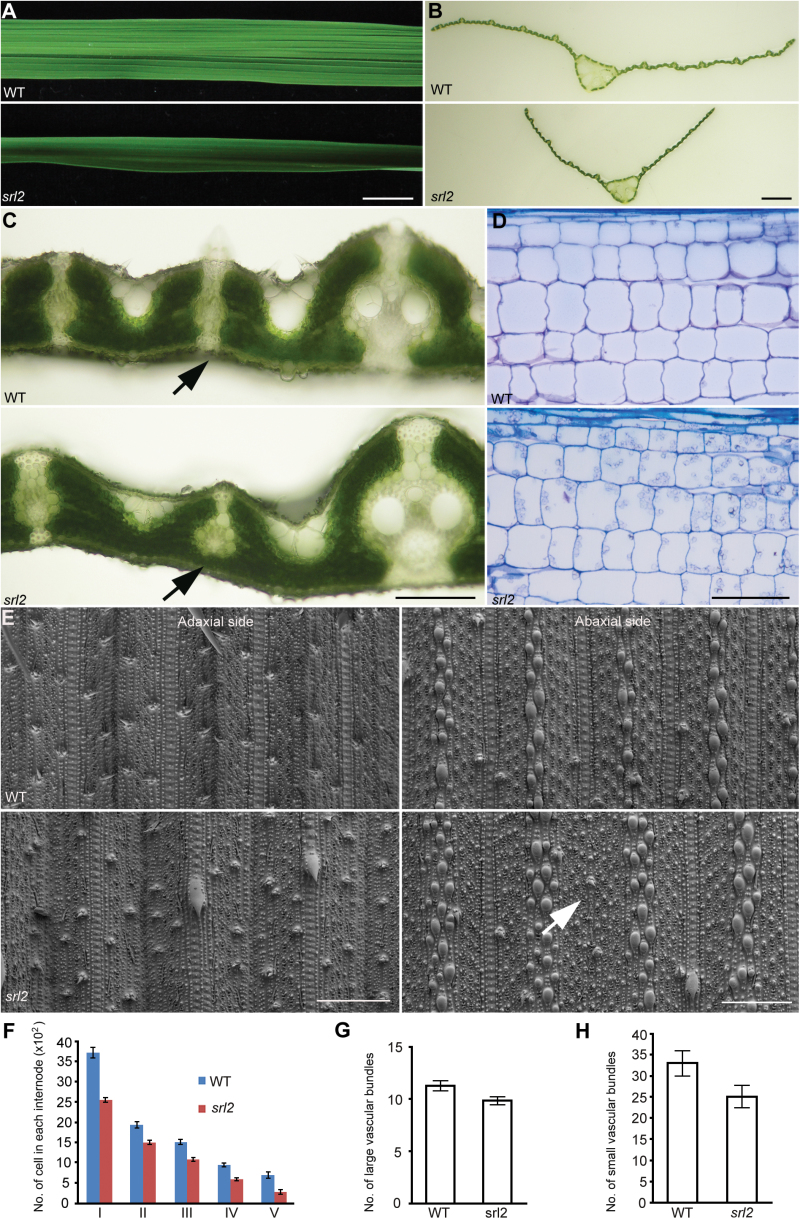
The *srl2* mutant has incurved leaves, with a deficiency of sclerenchymatous cells on the abaxial side. (A–C) Defective sclerenchymatous cells on the abaxial side of *srl2* leaves. The defect is mainly present in the small veins where the curl occurs. (D) Longitudinal sections of wild-type and *srl2* internodes. (E) Scanning electron micrograph of the adaxial and abaxial surfaces of wild-type and *srl2* leaves. (F) Number of cells in each internode (mean of four culms ±SD). Quantification of the large veins (LV) (G) and small veins (SV) (H) in leaves of wild-type and *srl2* plants (mean of six leaves ±SD). The arrows in (C) indicate the difference in abaxial sclerenchymatous cells between the wild type and *srl2*. The arrow in (E) indicates the absence of dumb-bell-shaped silica cells in the abaxial side of *srl2* leaves. Scale bars=1cm (A), 1mm (B), 100 μm (C, D) and 150 μm (E).

As mentioned above, the *srl2* mutant had semi-rolled leaves. To determine the cause of the rolled leaf phenotype, we performed anatomical observations of leaves. In contrast to the wild type, cross-sections of mature *srl2* leaves displayed altered mesophyll cell differentiation and distribution. The mutant did not form abaxial sclerenchymatous cells in the small veins of the lateral region (Fig. 4A–C) where the leaf began to roll; however, the cellular organization in the midrib region and large veins was similar to that of the wild type. In addition, not all small veins lacked sclerenchymatous cells in the abaxial side, statistical analysis indicated that approximate 20% of small veins per leaf (20.37±2%, *n*=7) lacked sclerenchymatous cells (Supplementary Table S7).

To confirm our results further, we performed an ultrastructural observation of the leaf epidermis by scanning electron microscopy (SEM). Epidermal cells in wild-type rice leaves are arranged regularly in rows that are parallel to the midrib. At the centre of the leaf surface (on both the adaxial and abaxial sides) where a vascular bundle occurs, dumb-bell-shaped silica cells lay side-by-side. These dumb-bell-shaped silica cells form one file directly over or under a small vascular bundle. The adaxial side of the leaf epidermis in *srl2* was comparable with that of the wild type. In the abaxial side of *srl2* leaf epidermis, however, the file formed by dumb-bell-shaped silica cells was absent under some small vascular bundles (Fig. 4E). These results further confirm that the differentiation of some perivascular sclerenchyma cells in the abaxial sides of *srl2* leaves was defective, indicating that the absence of sclerenchymatous cells in the abaxial side of leaves may account for the rolled leaf phenotype. A similar defect in sclerenchymatous cell development in the abaxial sides of leaves was also observed in the *sll1*/*rl9* mutant (Yan *et al.*, 2008; Zhang *et al.*, 2009), and this defect was also considered to produce rolled leaves in *sll1*/*rl9*.

In addition, another major phenotype of *srl2* is moderate dwarfism at all stages of growth and development. At the mature stage, the height of mutant plants was reduced by more than 30% (Fig. 1B). To determine the cause of the dwarf phenotype in *srl2* plants, we examined the anatomical features of cells in mutant and wild-type culms. First, we compared the internode cell size in both the transverse and longitudinal directions in mutant versus wild-type plants and found that these values were not significantly different (Fig. 4D). We also compared the cell number in the longitudinal direction (which contributes to the length of each internode) between *srl2* and wild-type plants. The cell numbers in each internode of *srl2* were only 40–70% those of the wild type (Fig. 4F; Supplementary Table S8). Furthermore, examining cross-sections of leaves and leaf sheaths helped confirm that the thin culms and narrow leaves resulted from reduced cell numbers, as determined by counting the number of vascular bundles (Fig. 4G, 4H; Supplementary Table S9). Therefore, reduced cell numbers might be the main factor contributing to the small size of the mutant plants.

### Expression patterns of *SRL2*

**Fig. 5.**
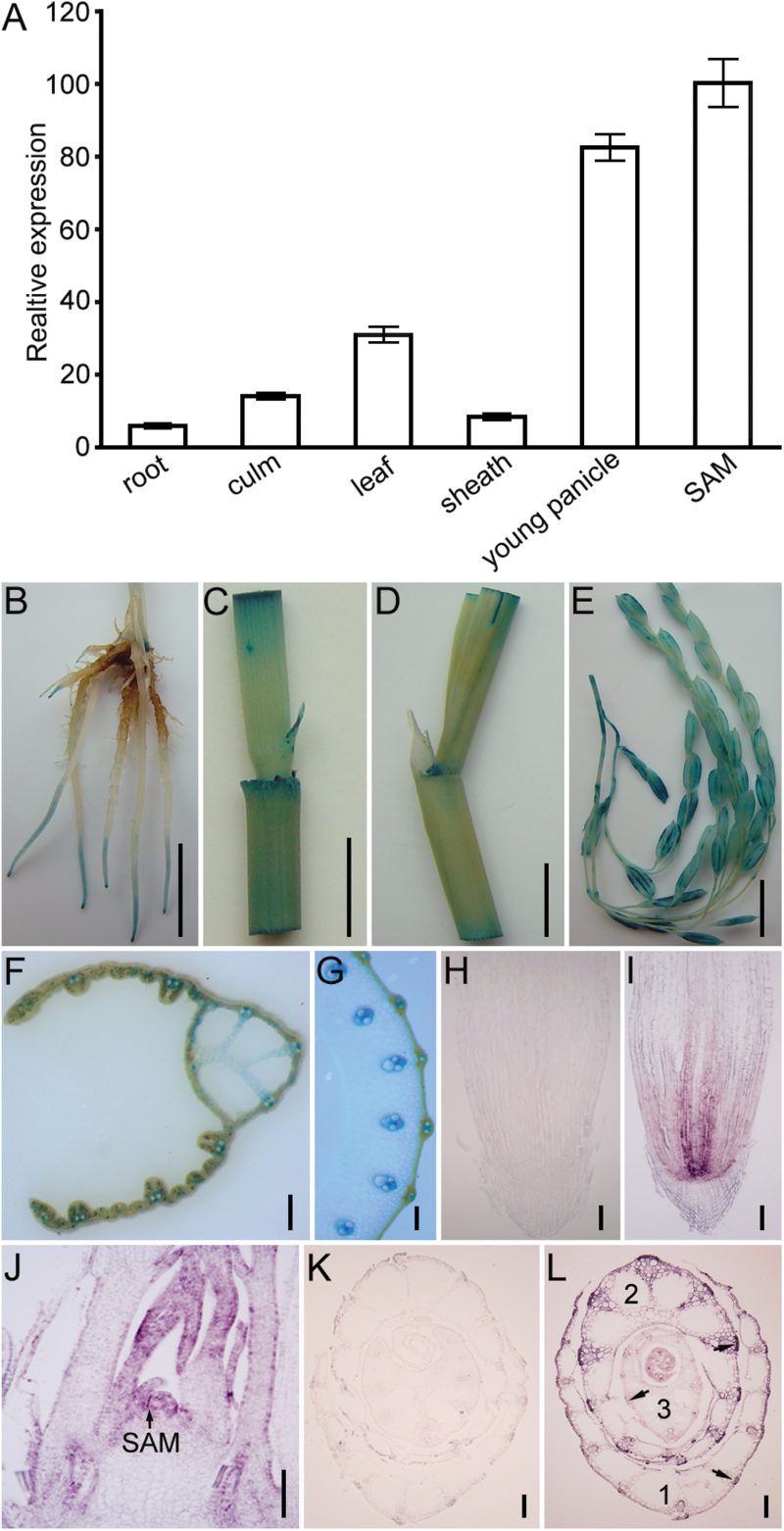
Expression pattern of *SRL2*. (A) qRT-PCR analysis of *SRL2* in various rice tissues. The expression levels are percentages of that of *Ubiquitin*. Values are means ±SD of triplicate assays. (B–G) GUS patterns in various organs of *Pro*_*SRL2*_ *-GUS* transgenic plants, showing GUS signals in root tips (cell division zone) (B), culm (C), leaf sheath (D), and young panicle (E). Hand-cut section of leaf (F) and culm (G) tissue. (H–L) *In situ* hybridization analysis of *SRL2* expression in root and SAM tissue. (H, K) *In situ* hybridization of control tissue using a sense probe. *SRL2* transcript was detected in root tips (cell division zone) (I), SAM (J), and the leaf sheath abaxial cell layer and vascular bundles (L). The arrows in (J) and (L) indicate the *in situ* hybridization signal. Scale bars=1cm (B–E), 100 μm (F–L).

We used quantitative real-time RT-PCR (qRT-PCR) analysis to investigate the expression of *SRL2* in various tissues, including root, mature leaf, internode (culm), and leaf sheath tissue (Fig. 5A). Strong expression of *SRL2* was detected in the shoot apical meristem (SAM) and young panicles before heading (Fig. 5A), suggesting that *SRL2* is highly expressed in rapidly growing tissues.

To assess the expression patterns comprehensively, we generated transgenic rice plants expressing the *GUS* reporter gene driven by the *SRL2* putative promoter (*Pro*_*SRL2*_ *-GUS*). β-glucuronidase (GUS) histochemical staining of transgenic plants demonstrated that *SRL2* was preferentially expressed in root tips (cell division zone) (Fig. 5B). In the aerial parts of plants, GUS staining was also visualized in all tissues, including culms (Fig. 5C), leaf sheaths (Fig. 5D), and young panicles (Fig. 5E) which is consistent with the results of qRT-PCR. Hand-cut sectioning of mature leaf blades and leaf sheaths further demonstrated that GUS was mainly expressed in vascular bundles (Fig. 5F, G; Supplementary Fig. S6).

To confirm that *SRL2* is preferentially expressed in rapidly growing tissues, we performed a more detailed characterization of *SRL2* transcript levels in root and SAM tissues by mRNA *in situ* hybridization. Hybridization signals were clearly expressed in root tip cells undergoing cell division (Fig. 5I), especially in the region that differentiates into the stele or vascular cylinder. Similar results were obtained for SAM tissue (Fig. 5J). Hybridization signals were not detected in sections probed with sense-strand *SRL2* RNA, which was used as a negative control (Fig. 5H).

We also examined the spatial and temporal localization of *SRL2* during leaf development by *in situ* hybridization analysis. Cross-section analysis of the shoot apex showed that *SRL2* was highly expressed in the leaf sheath abaxial cell layer and in vascular bundles, especially in sclerenchymatous cells that had undergone a certain degree of development. *SRL2* was dynamically expressed in the sclerenchymatous cell zone. As shown in Fig. 5L, the leaf sheath comprises three layers (layer 1–3, from the outside to the inside). *SRL2* mRNA signals were relatively weak in layer 1, significantly stronger in layer 2, and weak in layer 3 (Fig. 5L). However, very weak hybridization signals were detected in sections probed with sense-strand *SRL2* RNA (Fig. 5K).

In summary, *SRL2* appears to be ubiquitously expressed, although it is preferentially expressed in rapidly growing tissues and at specific stages of leaf development.

### *YAB4* and *YAB5* are dramatically down-regulated in *srl2*

Leaf development is regulated by several groups of genes such as auxin synthesis-related genes including *YUCCA* genes (Fujino *et al.*, 2008), auxin transport-associated genes (Benkova *et al.*, 2003), and *YABBY* genes (Dai *et al.*, 2007; Stahle *et al.*, 2009; Sarojam *et al.*, 2010; Tanaka *et al.*, 2012), as well as other leaf development-related genes. To gain insights into the *SRL2*-mediated mechanism in leaf organogenesis, we measured the mRNA levels of leaf development-associated genes in young leaves by qRT-PCR. We first examined the expression of auxin signal transduction, transport, and biosynthesis-related genes, such as *AUXIN RESPONSE FACTOR* (*ARF*), *PIN-FORMED* (*PIN*), and *YUC* family genes, revealing no obvious change in expression in *srl2*.

**Fig. 6.**
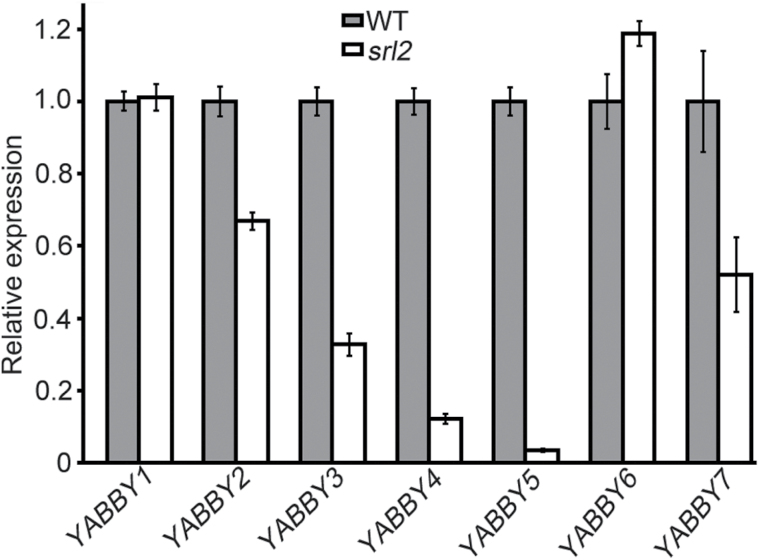
Expression of *YABBYs* in the wild type and *srl2*, using the *ubiquitin* gene as the internal control. Values are means ±SD of triplicate assays.

In rice, *SLL1*/*RL9* encodes a KANADI family protein involved in leaf abaxial cell development. Like *SRL2*, mutation of *SLL1*/*RL9* leads to defective development of sclerenchymatous cells in the abaxial sides of leaves (Yan *et al.*, 2008; Zhang *et al.*, 2009). These similar mutant phenotypes prompted us to investigate changes in *SLL1*/*RL9* expression in *srl2*, revealing that *SLL1*/*RL9* expression was unaffected in the mutant. Moreover, when we crossed *srl2* and *sll1* plants, *srl2 sll1* double mutant plants were obtained. Statistical analysis revealed that, compared with *srl2* and *sll1* single mutants, the *srl2 sll1* double mutant had more severely defective development of sclerenchymatous cells in the leaf abaxial side, together with a much more narrow leaf blade phenotype (Supplementary Fig. S7). These results suggest that *SRL2* and *SLL1*/*RL9* function in distinct pathways regulating the development of the abaxial side of the leaf. Finally, we examined the expression of other leaf development-related genes, such as *YABBY* family genes (Stahle *et al.*, 2009; Sarojam *et al.*, 2010; Tanaka *et al.*, 2012), *NAL1* (Qi *et al.*, 2008), *ND1* (Li *et al.*, 2009), and *RL14* (Fang *et al.*, 2012). Compared with the wild type, *YAB4* and *YAB5* were significantly down-regulated in *srl2* (5-fold and 27-fold, respectively), and *YAB2*, *YAB3*, and *YAB7* were also slightly down-regulated (Fig. 6). However, the expression levels of other *YABBY* genes, as well as *NAL1*, *ND1*, and *RL14* were not altered. These results suggest that the phenotypes of *srl2* may be related to the altered transcriptional activity of leaf development-related *YABBY* genes.

## Discussion

### Abnormal sclerenchymatous cells account for rolled leaves in *srl2*

Leaf rolling is a complex trait affected by multiple genes and even the same degree of leaf rolling can be caused by different genes (Hibara *et al.*, 2009; Zhang *et al.*, 2009; Li *et al.*, 2010; Zou *et al.*, 2011). Furthermore, differences in the expression of a single gene have been associated with differences in leaf rolling index (Zou *et al.*, 2011). In addition, the leaf rolling phenotype may be caused by more than one alteration in the structure of a particular cell type and may even be caused by changes in different or multiple cell types (Zou *et al.*, 2014). Bulliform cells, which are located between two vascular bundle ridges in parallel with the more adaxial-localized veins of grass leaves, play an important role in regulating leaf rolling in rice (Zou *et al.*, 2011; Xiang *et al.*, 2012). Sclerenchyma cells are important for maintaining the proper morphology in rice leaves. Sclerenchymatous cells, which originate from mesophyll cells through transdifferentiation, are lignified dead cells with thickened secondary cell walls. A deficiency in abaxial sclerenchyma cells results in adaxially rolled leaves due to the loss of force provided by sclerenchyma cells, whereas improper formation of sclerenchyma cells on the adaxial side of the leaf is associated with abaxial leaf rolling. *SHALLOT-LIKE1* (*SLL1*) controls the formation of abaxial sclerenchyma cells, and *SLL1* deficiency results in tight inwardly rolled leaves (Zhang *et al.*, 2009). Deficient adaxial sclerenchyma cells associated with increased parenchyma cells in vascular bundles in the *pci* mutants lead to outwardly rolled leaves (Zou *et al.*, 2014). Using SEM and histological analysis of *srl2* leaves, we found that *srl2* mutant leaves had abnormal sclerenchymatous cells. Since sclerenchymatous cells contribute continuous mechanical strength to maintain normal leaf shape, the lack of sclerenchymatous cells on the abaxial surface in curved regions of *srl2* leaves indicates that sclerenchyma plays a critical role in controlling leaf rolling in monocots and that *SRL2* functions in sclerenchyma cells formation.

### *SRL2* and *SLL1* may function in distinct pathways regulating abaxial-side development in leaves

*SLL1* encodes an SHAQKYF class MYB transcription factor involved in the transdifferentiation of mesophyll cells to sclerenchymatous cells through the modulation of programmed cell death and its deficiency leads to impaired formation of sclerenchymatous cells on the abaxial side of the leaf, resulting in extremely inwardly rolled leaves (Zhang *et al.*, 2009). In addition, *SLL1* is crucial to polarity formation and helps direct the development of the leaf abaxial cell layer (Zhang *et al.*, 2009). The defective leaf development in the *srl2* mutant is quite similar to that of *sll1* although they have varying degrees of leaf rolling (perhaps because not all small veins lacked sclerenchymatous cells in the abaxial sides of *srl2* leaves, and approximately 20% of small veins per leaf lacked sclerenchymatous cells). However, we did not find any obvious alteration in leaf polarity in *srl2*. Interestingly, the *srl2 sll1* double mutant exhibited a more severe defect in the development of sclerenchymatous cells in the abaxial side of the leaf, together with a more narrow leaf blade phenotype. Meanwhile, qRT-PCR revealed that the expression of *SLL1* was not affected in the *srl2* mutant. We propose that *SLL1* and *SRL2* act in two different ways to modulate abaxial development in leaves.

### *SRL2* is involved in multiple developmental processes

The expression of *YABBY* genes was altered in *srl2*. Genes in this small, plant-specific family encode putative transcription factors containing two conserved domains: a zinc-finger domain in the N-terminal region and a YABBY domain (helix-loop-helix motif) in the C-terminal region (Siegfried *et al.*, 1999). *YABBY* genes play important roles in regulating diverse developmental processes such as the establishment of adaxial–abaxial polarity, lamina expansion, and floral organ development in eudicots (Tanaka *et al.*, 2012). *Arabidopsis thaliana* contains only six *YABBY* genes, namely, *FILAMENTOUS FLOWER* (*FIL*), *CRABS CLAW* (*CRC*), *INNER NO OUTER* (*INO*), *YAB2*, *YAB3*, and *YAB5*, which play distinct roles in development (Bowman and Smyth, 1999; Sawa *et al.*, 1999; Siegfried *et al.*, 1999; Villanueva *et al.*, 1999). Rice contains eight *YABBY* genes, including *OsYABBY1*, *OsYABBY2*, *OsYABBY3*, *OsYABBY4*, *OsYABBY5*, *OsYABBY6*, *OsYABBY7*, and *DROOPING LEAF (DL*) (Toriba *et al.*, 2007). *DL*, a rice orthologue of CRC has been well characterized. Loss-of-function of *DL* causes the homeotic transformation of carpels into stamens in the flower and loss of the midrib in the leaf, suggesting that *DL* regulates carpel specification and midrib formation in rice (Nagasawa *et al.*, 2003; Yamaguchi *et al.*, 2004; Ohmori *et al.*, 2008; Ohmori *et al.*, 2011). *OsYABBY1*, which is similar to Arabidopsis *YAB2*, is expressed in putative precursor cells of a specific cell type, such as sclerenchyma (Juarez *et al.*, 2004; Toriba *et al.*, 2007). *OsYABBY5* (*TOB1*) is involved in lateral organ development and maintaining meristem organization in the rice spikelet. Mutation of this gene leads to pleiotropic phenotypes in spikelets, such as the formation of a cone-shaped organ instead of a lemma or palea, the development of two florets in a spikelet, and premature termination of the floret meristem, in addition to reduced growth of the lemma or palea and elongation of the awn (Tanaka *et al.*, 2012). In the *srl2* mutant, the expression of some *YABBY* genes was altered. For example, *YAB2*, *YAB3*, *YAB4*, *YAB5*, and *YAB7* were down-regulated, but *YAB6* was up-regulated. In particular, the expression of *YAB5* was significantly down-regulated (27-fold) in *srl2*, but this mutant did not exhibit the same phenotype as the *tob1* mutant which might be due to the effect of the altered expression of more than one *YABBY* gene. These results suggest that the phenotype of *srl2* may be related to the altered transcriptional activity of *YABBY* genes. All of these results demonstrate that *SRL2* has multiple effects on plant development.

### Mutants represent an excellent genetic resource for ideal plant architecture breeding in rice

Improving grain yield is the ultimate goal of rice breeding. As leaves represent the major photosynthetic organ of plants, optimal leaf structure is important for maximizing light capture and for efficient gas exchange. The moderately rolled leaf is regarded as a critical component of the ideal rice phenotype as it is important for improving photosynthetic efficiency and grain yields by maintaining the erectness of leaves and minimizing shadowing between leaves (Yuan, 1997; Wu, 2009). The shortage of new germplasm resources with important agronomic features is a major barrier to the development of cultivars with higher yields (Wang and Li, 2005). Therefore, it is critical to identify new mutants with moderately rolled leaves and to isolate genes related to these features. In the present study, we performed an in-depth characterization of a semi-rolled leaf mutant which may be useful for breeding rice with an ideal plant architecture.

## Supplementary data

Supplementary data can be found at *JXB* online.

Table S1. Primers used in map-based cloning of *SRL2.*

Table S2. Primers used for *in situ* hybridization analysis.

Table S3. Primers used in real-time qRT-PCR analysis.

Table S4. The width of the leaf blade in both the wild type and *srl2*.

Table S5. The height of plants in both the wild type and *srl2*.

Table S6. The length of internode in both the wild type and *srl2*.

Table S7. The number of abnormal small vascular bundles in *srl2*.

Table S8. The number of cells in each internode in both the wild type and *srl2*.

Table S9. The number of large vascular bundles and small vascular bundles in both the wild type and *srl2*.

Figure S1. Morphological comparison of wild-type and *srl2* panicles.

Figure S2. Deduced amino acid sequences of SRL2 and the changes in amino acids caused by mutations.

Figure S3. RNAi analyses.

Figure S4. Subcellular location of the SRL2–GFP fusion protein in onion epidermal cells.

Figure S5. Western blot assays.

Figure S6. Hand-cut section of a *Pro*_*SRL2*_ *-GUS* transgenic rice leaf.

Figure S7. Characteristics of the *srl2 sll1* double mutant.

Supplementary Data
