# Supplementary material for: Semi-Rolled Leaf2 modulates rice leaf rolling by regulating abaxial side cell differentiation
Source: J Exp Bot. 2016 Feb 11;67(8):2139–50. doi: 10.1093/jxb/erw029 (PMC4809286; doi:10.1093/jxb/erw029)
Supplement: Supplementary Data [file supp_67_8_2139__index.html]

 Semi-Rolled Leaf2 modulates rice leaf rolling by regulating abaxial side cell differentiation — Semi-Rolled Leaf2 modulates rice leaf rolling by regulating abaxial side cell differentiation — Supplementary Data 

# *Semi-Rolled Leaf2* modulates rice leaf rolling by regulating abaxial side cell differentiation

## Supplementary Data

Data files

- supplementary\_figures\_S1\_S7\_Tables\_S1\_S9.pdf - Supplementary Data
