# Supplementary material for: Semi-Rolled Leaf2 modulates rice leaf rolling by regulating abaxial side cell differentiation
Source: J Exp Bot. 2016 Feb 11;67(8):2139–50. doi: 10.1093/jxb/erw029 (PMC4809286; doi:10.1093/jxb/erw029)
Supplement: Supplementary Data [file supp_erw029_supplementary_figures_S1_S7_Tables_S1_S9.pdf]

Supplementary Data

Supplementary Fig. S1

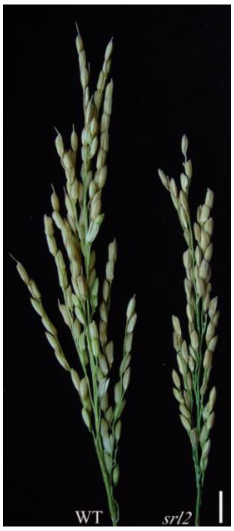

Supplementary Fig. S1. Morphological comparison of wild-type and *srl2* panicles. Scale bars = 1 cm.

Supplementary Fig. S2

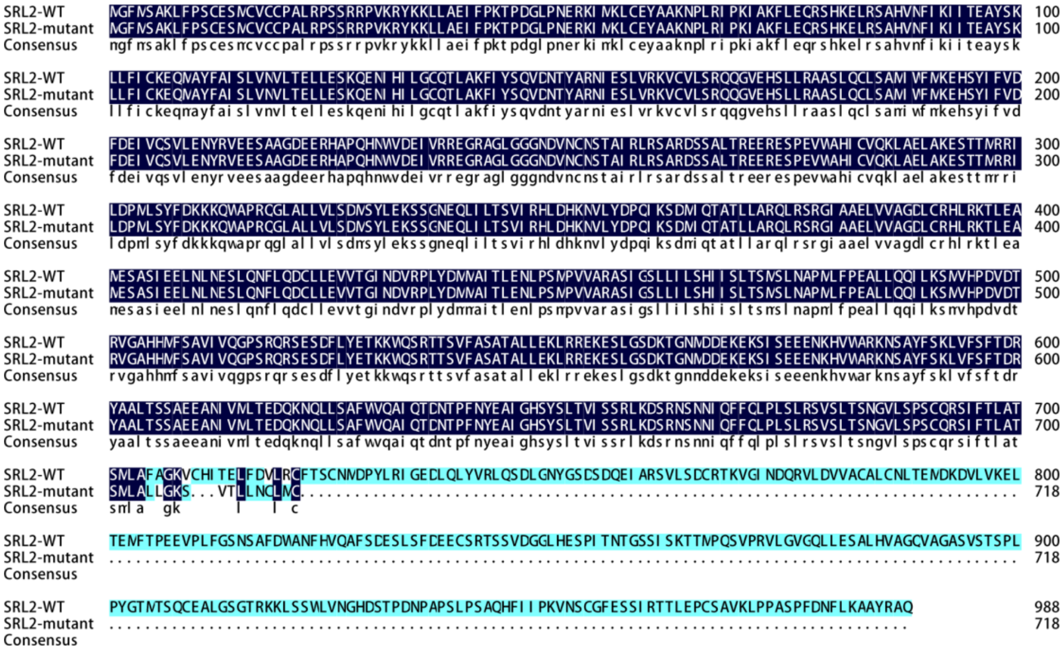

Supplementary Fig. S2. Deduced amino acid sequences of SRL2 and the changes in amino acids caused by mutations.

Supplementary Fig. S3

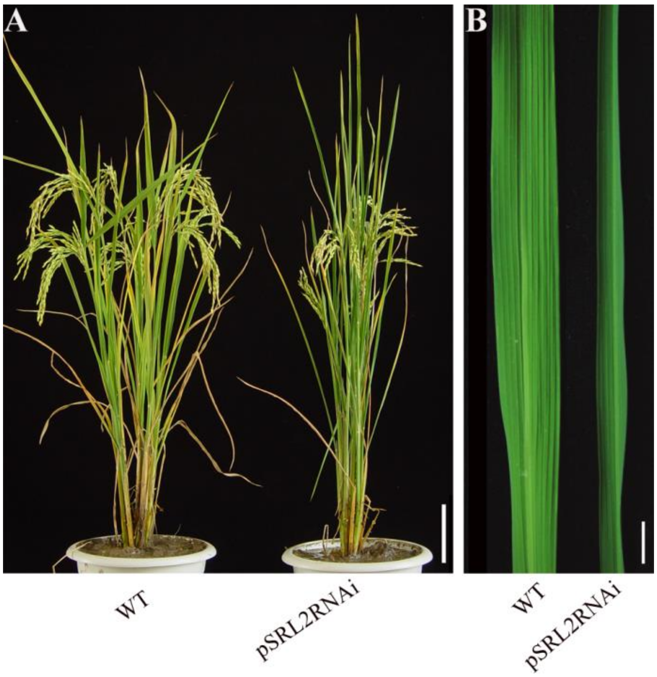

Supplementary Fig. S3. RNAi analyses. Scale bars = 10 cm (A) and 1 cm (B).

Supplementary Fig. S4

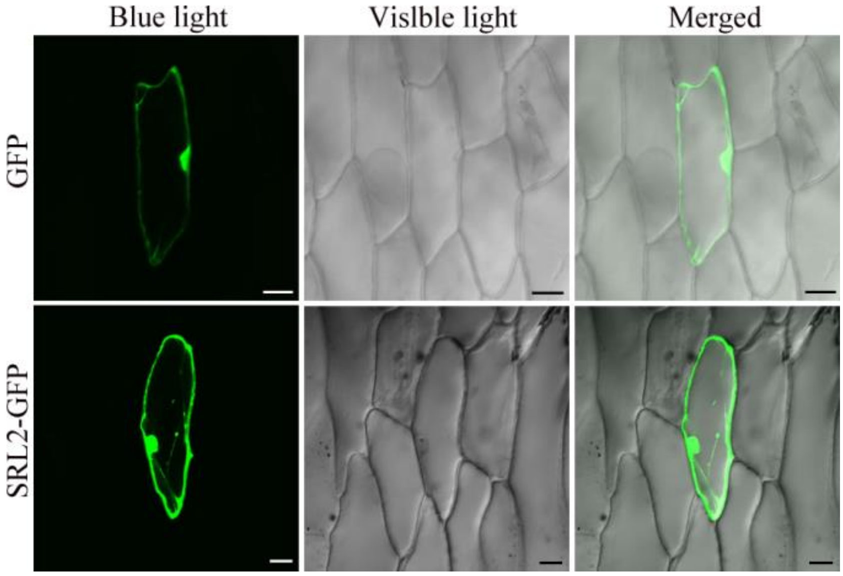

Supplementary Fig. S4. Subcellular location of the SRL2-GFP fusion protein in onion epidermal cells. Onion epidermal cell expressing free GFP showed fluorescence in the nucleus and cytoplasm. Onion epidermal cell expressing SRL2-GFP showed fluorescence in the nucleus and cytoplasm. Scale bars = 50 μm.

Supplementary Fig. S5

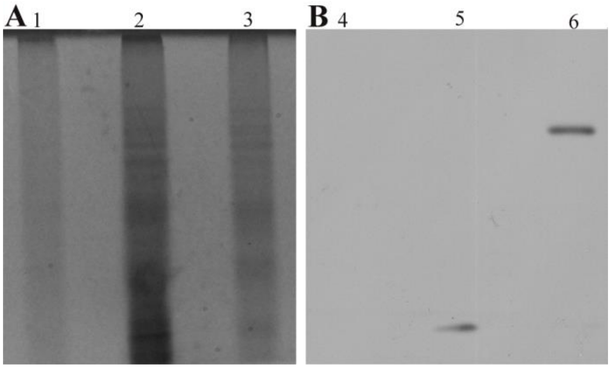

Supplementary Fig. S5. Western blot assays. (A) An SDS–PAGE gel stained with Coomassie Brilliant Blue. (B) Western blotting of the GFP and SRL2-GFP using anti-GFP antibody. Line 1, 4: Total protein from onion epidermis as a negative control; Line 2, 5: Total protein from onion epidermis expressing free GFP; Line 3, 6: Total protein from onion epidermis expressing SRL2-GFP.

Supplementary Fig. S6

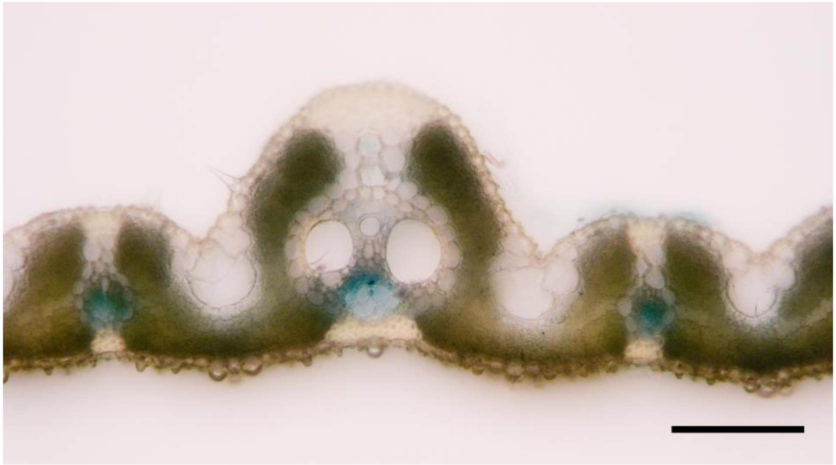

Supplementary Fig. S6. Hand-cut section of $\textit{Pro}_{\textit{SRL2}}$-*GUS* transgenic rice leaf. Scale bars = 100 μm.

Supplementary Fig. S7

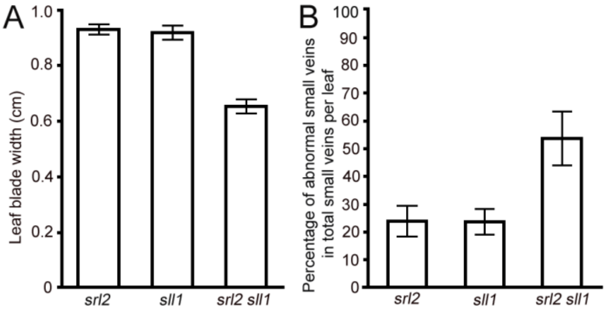

Supplementary Fig. S7. Characteristics of the *srl2 sll1* double mutant. (A) The leaf width of leaf blade in *srl2*, *sll1* and *srl2 sll1* double mutant. (B) The percentage of abnormal small veins in *srl2*, *sll1* and *srl2 sll1* double mutant.

Table S1: Primers used in map-based cloning of *SRL2*

Marker	Primers	Fragment size (bp)
S1	F 5'-TGTATGAAGAGGAGTGGAGC-3'	100/115
	R 5'-GGTTAATTGGTTTGGTTGGC-3'	
S2	F 5'-CTCCCAATTGGTGGGGTATG-3'	120/100
	R5'-ACTTAACACCATTACCCTGC-3'	
S3	F 5'-CGAAACCTCGTCAGTTCGAG-3'	130/115
	R 5'-TTGGAGGGCAATACGGGGAT-3'	
S4	F 5'-AGGAAGGTGCCTCTCTTCGA -3'	140/120
	R 5'-CCTGGTTTAGGAGCTTCTCC-3'	
S5	F 5'-CATGAGTATGTCAATGCTGC-3'	100/120
	R 5'-TCTCTTGCTAATATCTAGCC -3'	
S6	F 5'-TACGGTTGCACGGTCAAAGA -3'	100/120
	R 5'-TCGTGTGCATCAAGTTGAGG-3'	

Table S2: Primers used for in situ hybridization analysis

Primer name	Primer pairs
SRL2 T7	GAAGCTCTCAAGCTGGCTTG
SRL2 sp6	CAATATGGTCATACATGGTC

Table S3: Primers used in real-time qRT-PCR analysis

Gene	Primer pairs
SRL2	TCCATCTGCGCAGCATTTCA
	CTACTGGGCACGATATGCAG
YABBY1	TGGTGAATGTGCCAAACAAT
	GCTTGGTGAAAAGGAGCAAG
YABBY2	AATTTTCGCGGTCAGTGTTC
	AGTTTTCGCGGAAGCTCATA
YABBY3	ATCAAGGACGAAATCCAACG
	GGCATCAGTCCAAAGTGGAT
YABBY4	ATCACATCAAAGGGGACCAA
	CGAGTACGCAATGGCCTTAT
YABBY5	GAGCCTAATGACCGAACAGG
	CTCTGCCGCTTCTCTGAAGT
YABBY6	TCTGTTCATCACTGGCTTCG
	CGTGTTGCAGAAGTTGCAGT
YABBY7	CACAGCTGCTAAAAACTGGG
	CTGGACAGCTACAACTTGGA
Ub	GTCTGATCTTCGCTGGCAAGCAGC
	GCATACTGCTGTCCCACAGGAAACTG

Table S4: The width of leaf blade in both wild type and *srl2* (mm)

	WT		*srl2*	
	before stretched	after stretched	before stretched	after stretched
1	11	11.8	6	8
2	11.5	12	4	8
3	12	12	6	8.5
4	13	13	6	9
5	12.5	12.5	4	7
6	12	12	6	9
7	12	12	5.5	8
8	12	12	6	8
9	12.5	13	4	8.2
10	12	12	4	8
11	13	13	5	8
12	11.5	11.5	5	8.5
13	12.5	13	5	8
14	12	12.8	4	8
15	12	12	3.5	7
16	13	14	5	9
17	11.8	12	5	8.5
18	14	14	5	9
19	12.5	13	5	8
20	11.5	12.5	5	8
21	11	12	5	8
22	12	13	4	7
23	13	13	3	8
24	11	11.5	4	8
25	12	12.5	4	7.5
26	12	13	4	7
27	12.5	13.5	5	8.5
28	12	13	5	8
29	11.5	12	5	8.5

1-29: different rice plants.

Table S5: The height of plants in both wild type and *srl2* (cm)

	1	2	3	4	5	6	7	8	9	10
WT	78.2	79.5	81.3	78.6	78.4	81.2	78.1	79.2	82.1	79.2
srl2	56.1	55.2	57.2	54.3	56.5	56.7	57.4	55.1	55.2	54.3

1-10: different rice plants.

Table S6: The length of internode in both wild type and *srl2* (cm)

	WT					*srl2*				
	I	II	III	IV	V	I	II	III	IV	V
1	27.7	12.8	8.5	6.7	2.2	17.8	7.5	4.1	2.5	1.5
2	27	12	8.2	6.8	2.1	19	7	4	3.1	1.3
3	27.6	13	8.5	6.4	2.4	17.5	7	4.2	3	1.2
4	26.1	11	7.2	5.8	2.3	16.5	7.2	4	3	1.2
5	26.8	12	8.8	6	2.2	17	8	3.8	2.5	1.1
6	27.5	12.1	8.1	6.5	1.8	20	8.5	5.5	3.5	1.5
7	27	13.2	8.5	6.5	2.2	18	8	5.6	2.5	1.1
8	28.5	13.2	8.5	6.2	2.4	17.5	7.2	4.2	2	0.9
9	26.8	12.5	7.2	6.4	2.5	18	7.8	3	1.5	1.2
10	28.5	12.4	7.8	6.5	2.5	17.5	7.2	5.5	3.5	0.9

1-10: different rice plants. I–V: the 1st to 5th internodes.

Table S7: The number of abnormal small vascular bundles in *srl2*

	No. of abnormal SV	No. of normal SV	No. of total SV
A	5	17	22
B	5	23	28
C	5	18	23
D	6	22	28
E	5	18	23
F	5	23	28
G	5	21	26

A-G: different rice plants. SV: small vascular bundles

Table S8: The number of cell in each internode in both wild type and *srl2*

	WT					*srl2*				
	I	II	III	IV	V	I	II	III	IV	V
1	3523	1991	1545	924	560	2591	1385	1045	555	240
2	3796	1845	1436	899	768	2456	1503	1080	600	280
3	3633	1874	1508	927	731	2501	1488	1150	515	300
4	3881	2008	1568	1001	677	2602	1550	1050	650	326

1-4: different rice plants. I–V: the 1st to 5th internodes.

Table S9: The number of large vascular bundles and small vascular bundles in both wild type and *srl2*

	WT		*srl2*	
	No. of LV	No. of SV	No. of LV	No. of SV
1	12	38	10	22
2	11	33	10	28
3	12	34	9	23
4	11	34	10	28
5	11	33	10	23
6	11	28	10	28

1-6: different rice plants; LV: large vascular bundles; SV: small vascular bundles.
